# Supplementary material for: Identifying potential sites for rainwater harvesting ponds (embung) in Indonesia’s semi-arid region using GIS-based MCA techniques and satellite rainfall data
Source: PLoS One. 2023 Jun 7;18(6):e0286061. doi: 10.1371/journal.pone.0286061 (PMC10249980; doi:10.1371/journal.pone.0286061)
Supplement: S2 File — (DOCX) [file pone.0286061.s003.docx]

13 March 2023

Number : B-13.13/PPIG-BIG/IIG.01.04/3/2023

Characteristic : Ordinary

Attachment : 1 (one) document

Subject : Terms & Conditions of Usage Notice

Peta dari Ina-Geoportal

Dear Mr. Yulius P.K. Suni

We provide the following information in response to the email sent through the NSDI helpdesk regarding Ina-request Geoportal's for a map usage permit (https://tanahair.indonesia.go.id/).

- Basic Geospatial Information (IGD) contained in Ina-Geoportal is a product of the Geospatial Information Agency (BIG) and the copyright is owned by BIG. On the condition that BIG is cited in the reference, users are allowed and free to download, distribute, adapt, or develop modified versions of the IGD on the Ina-Geoportal website. Users are prohibited from selling any data they get from this portal.
- Citation example: Badan Informasi Geospasial Republik Indonesia, 2015-2019. Peta Rupabumi Digital Indonesia. Bogor, Jawa-Barat. Accessed from: <http://tanahair.indonesia.go.id/portal-web/>.

This is explained in order for it to be applied appropriately.

Head of Center for Management and Dissemination of Geospatial Information,

Rachman Rifai

CC to:

1. Deputy for Geospatial Information Infrastructure
