# Supplementary material for: Identifying potential sites for rainwater harvesting ponds (embung) in Indonesia’s semi-arid region using GIS-based MCA techniques and satellite rainfall data
Source: PLoS One. 2023 Jun 7;18(6):e0286061. doi: 10.1371/journal.pone.0286061 (PMC10249980; doi:10.1371/journal.pone.0286061)
Supplement: S3 File — (PDF) [file pone.0286061.s004.pdf]

BADAN INFORMASI GEOSPASIAL
(B I G)

Jl. Raya Jakarta – Bogor KM 46 Cibinong Bogor 16911
Telepon (021) 875 2062-2063, Faksimile (021) 875 2064 PO. Box 46 CBI
Website: http://www.big.go.id

Nomor	:	B-13.13/PPIG-BIG/IIG.01.04/3/2023	13 Maret 2023
Sifat	:	Biasa	
Lampiran	:	1 (satu) berkas	
Hal	:	Surat Pemberitahuan Syarat dan Ketentuan Penggunaan Peta dari Ina-Geoportal	

Yth. Sdr. Yulius P.K. Suni
di tempat

Berdasarkan email yang masuk melalui helpdesk NSDI terkait permohonan izin penggunaan peta dari Ina-Geoportal (https://tanahair.indonesia.go.id/) maka kami sampaikan sebagai berikut:

- Informasi Geospasial Dasar (IGD) yang terdapat dalam Ina-Geoportal adalah produk Badan Informasi Geospasial (BIG) dan hak ciptanya dimiliki oleh BIG. Pengguna diijinkan dan dibebaskan untuk mengunduh, mendistribusikan, mengadaptasi atau membuat turunan IGD yang ada dalam website Ina-Geoportal, dengan syarat mencantumkan sumber informasi/data berasal dari BIG. Pengguna tidak diperkenankan untuk memperjualbelikan kembali segala data yang diperoleh dari portal ini.
- Contoh sitasi: Badan Informasi Geospasial Republik Indonesia, 2015-2019. Peta Rupabumi Digital Indonesia. Bogor, Jawa-Barat. Diakses dari : http://tanahair.indonesia.go.id/portal-web/.

Demikian disampaikan agar dapat digunakan sebagaimana mestinya.

Kepala Pusat Pengelolaan dan
Penyebarluasan Informasi
Geospasial,

Rachman Rifai

Tembusan:

1. Deputi Bidang Infrastruktur Informasi Geospasial

Dokumen ini telah ditandatangani secara elektronik yang diterbitkan oleh Balai Sertifikasi Elektronik (BSrE), BSSN
