# Supplementary material for: Identifying potential sites for rainwater harvesting ponds (embung) in Indonesia’s semi-arid region using GIS-based MCA techniques and satellite rainfall data
Source: PLoS One. 2023 Jun 7;18(6):e0286061. doi: 10.1371/journal.pone.0286061 (PMC10249980; doi:10.1371/journal.pone.0286061)
Supplement: Attachment — Submitted filename: Response to Reviewers.docx [file pone.0286061.s005.docx]

**Rebuttal Letter**

This letter responds to each point highlighted by the academic editor and reviewers.

**Responds to academic editor:**

1. Please ensure that your manuscript meets PLOS ONE's style requirements, including those for file naming.

**Response**: Our manuscript complied with PLOS ONE’s style guidelines.

2. We note that you have indicated that data from this study are available upon request. PLOS only allows data to be available upon request if there are legal or ethical restrictions on sharing data publicly.

**Response**: The data mentioned are raster maps and satellite rain data that were used in the analysis.

3. We note that Figures 1 to 5 in your submission contain [map/satellite] images which may be copyrighted. All PLOS content is published under the Creative Commons Attribution License (CC BY 4.0), which means that the manuscript, images, and Supporting Information files will be freely available online, and any third party is permitted to access, download, copy, distribute, and use these materials in any way, even commercially, with proper attribution. For these reasons, we cannot publish previously copyrighted maps or satellite images created using proprietary data, such as Google software (Google Maps, Street View, and Earth). For more information, see our copyright guidelines: <http://journals.plos.org/plosone/s/licenses-and-copyright>. We require you to either (1) present written permission from the copyright holder to publish these figures specifically under the CC BY 4.0 license, or (2) remove the figures from your submission.

**Response:** The figures were derived from Ina-Geoportal website, Indonesia official web for spatial data managed by Geospatial Information Agency (BIG). The website is open source. For the purpose of publishing information from the BIG website, we have received a license from Ina-Geoportal. Although BIG made a license in accordance with BIG standard, BIG did not complete the form we submitted. The license is in Bahasa Indonesia but the translation is available.

- Basic Geospatial Information (IGD) contained in Ina-Geoportal is a product of the Geospatial Information Agency (BIG) and the copyright is owned by BIG. On the condition that BIG is cited in the reference, users are allowed and free to download, distribute, adapt, or develop modified versions of the IGD on the Ina-Geoportal website. Users are prohibited from selling any data they get from this portal.
- Citation example: Badan Informasi Geospasial Republik Indonesia, 2015-2019. Peta Rupabumi Digital Indonesia. Bogor, Jawa-Barat. Accessed from: http://tanahair.indonesia.go.id/portal-web/.

**Responds to reviewers' comments:**

Reviewer #1

1- The abstract is generally well written but some modifications are suggested. Authors are requested to add the information how the criteria are selected?

**Response:** The criteria for determining the location of the reservoir refer to the FAO and Indonesia’s small ponds guideline. This sentence has been added in abstract.

2- The future recommendations should also be provided in abstract section

**Response**: It is recommended that decision-makers and managers of water resources employ this quick and cheap technique since it offers reasonable alternative sites for RWH. This sentence has been added in abstract.

3- Line 67 Please write Geographic Information System (GIS).

**Response**: Revision has been made accordingly in ‘Manuscript’ line 70.

4- The authors did not mention about the selections of criteria.

**Response**: Selections criteria were discussed in Line 182 – 191.

5- Map of the study area is not clear. Authors are requested to enhance the resolution and size of the figure.

**Response**: A new map with good resolution has been added.

6- It would be better to add the rainfall and temperature maps in the manuscript, so that the readers clearly understand the distribution pattern of rainfall, temperature, runoff etc.

**Response**: We only make a map of rainfall distribution in the watershed in **Fig. 2**, while temperature data is not available.

7- Line 79 Support this sentence with proper references such as doi:10.1088/1757-899X/881/1/012170; <https://doi.org/10.1155/2021/5580286>

**Response**: The reference has been added in 'Manuscript’ Line 82.

8- Line 87 -88 please write the longitudes and latitudes in correct forms such as 123⁰37′23.3″.

**Response**: Revision has been made accordingly in ‘Manuscript’ Line 90-91.

9- Data collection are not well captured hence suffering from many shortcomings.

**Response**: Data collection were revised and discussed in ‘Manuscript’ Line 111-119.

10- Please add more information about Land cover and land use, how the authors is conducted, which method has been on---etc.

**Response**: Revision has been made. In this study, land use and land cover are based on official and opensource detail map of Indonesia 2019, The map was developed by Indonesia Geospatial Information authority (BIG). Based on USGS Land Use and Land Cover, the study area was divided into seven different categories of land use and cover, including water, built-up land, barren land, forest land, shrubland, herbaceous natural, and cultivated land. These sentences are part of discussion on Land Use and Land Cover.

11- Table 1. Correct Area km2.

**Response**: Revision has been made accordingly.

12- Proper justification on the selection of the binary technique and the Weighted Linear Combination (WLC) in the Geographic information system (GIS), should be reported with proper referencing.

**Response:** Revision has been made. Three approaches were used to conduct multi-criteria analysis: the binary technique proposed by Eastman (1999) as cited by [37], the weighted linear combination (WLC) [38,39]…

13- Proper justification on the selection of the AHP should be reported with proper referencing.

**Response:** Revision has been made accordingly. … and the analytical hierarchy procedure (AHP) proposed first by Saaty (1980) as mentioned by [40].

14- Line 227-235 the rainfall runoff relationship is discussed in many references please, add more references such as <https://doi.org/10.18280/ijdne.150511>; https://doi.org/10.1007/s12518-021-003; <https://doi.org/10.18280/ijdne.150318>;

**Response**: Revision has been made accordingly in ‘Manuscript’ Line 170-180 and Line 256-263. Equation for flow discharge calculation has been changed to SCS and CN method as discussed by aforementioned references.

15- Line 287 please correct Area km2

**Response**: Revision has been made accordingly.

16- About the use of the WLC. First, I ask the authors to specify that the operation to refer all criteria to the same scale must be referred as ‘normalisation’. The authors must also specify that they used a raster model.

**Response**: Revision has been made. We discuss this issue in the ‘Manuscript’ Line 213-216. Map-based processing was used to process the nine criteria. All maps were formatted into a raster file (tif) format. Additionally, the nine criteria were applied to an overlay (multi-criteria analysis) method. The normalization technique is used to weight each criterion, with each criterion given the same weight [40].

17- Are there any sensitivity analyses conducted for the selected criteria?

**Response**: We are aware of the importance of sensitivity analysis, but we prefer not to employ it because the analysis' findings could be verified with a simple survey of potential RWH sites.

18- Authors are requested to add future recommendations for the study area.

**Response**: Recommendation has been added on Conclusion part (‘Manuscript’ Line 344-348). The combination of geospatial data, GIS, a multi-criteria analysis, and a field survey proved effective for the RWH site selection in a semi-arid region with limited data, especially on the first and second order streams. It is recommended that decision-makers and managers of water resources employ this quick and cheap technique since it offers reasonable alternative sites for RWH.

Reviewer #2

1. Provide reference/citations for the suitability/unsuitability of soils for reservoir (Table 2. Soil types in Liliba watershed).

**Response**: The references to soil types and soil suitability/unsuitability have been added to Table 2, which has been changed to Table 3.

2. Provide reference for the suitability/unsuitability of land covers for reservoir (Table 1. Land cover and land use statistical data).

**Response**: The references for suitability/unsuitability of land covers have been added to Tabel 1, which has been changed to Table 2.

3. Improve citations overall the paper.

**Response**: The citation has been corrected, and the proper references were applied.

4. Include high quality figures.

Response: Figures with poor quality have been replaced.

5. Include appropriate titles for all figures.

**Response**: Revision has been made.

6. Make border scale for all figures.

**Response**: Revision has been made.
